# Estimating the Impacts of Local Policy Innovation: The Synthetic Control Method Applied to Tropical Deforestation

**DOI:** 10.1371/journal.pone.0132590

**Published:** 2015-07-14

**Authors:** Erin O. Sills, Diego Herrera, A. Justin Kirkpatrick, Amintas Brandão, Rebecca Dickson, Simon Hall, Subhrendu Pattanayak, David Shoch, Mariana Vedoveto, Luisa Young, Alexander Pfaff

**Affiliations:** 1 Department of Forestry and Environmental Resources, North Carolina State University, Raleigh, NC, United States of America; 2 Sanford School of Public Policy, Duke University, Durham, NC, United States of America; 3 IMAZON, Amazon Institute of People and the Environment, Belém, Brazil; 4 TerraCarbon LLC., Charlottesville, VA, United States of America; 5 National Wildlife Federation, Washington, D.C., United States of America; 6 School of Forestry and Environmental Studies, Yale University, New Haven, CT, United States of America; 7 Geography Department, Clark University, Worcester, MA, United States of America; University of Vermont, UNITED STATES

## Abstract

Quasi-experimental methods increasingly are used to evaluate the impacts of conservation interventions by generating credible estimates of counterfactual baselines. These methods generally require large samples for statistical comparisons, presenting a challenge for evaluating innovative policies implemented within a few pioneering jurisdictions. Single jurisdictions often are studied using comparative methods, which rely on analysts’ selection of best case comparisons. The synthetic control method (SCM) offers one systematic and transparent way to select cases for comparison, from a sizeable pool, by focusing upon similarity in outcomes before the intervention. We explain SCM, then apply it to one local initiative to limit deforestation in the Brazilian Amazon. The municipality of Paragominas launched a multi-pronged local initiative in 2008 to maintain low deforestation while restoring economic production. This was a response to having been placed, due to high deforestation, on a federal “blacklist” that increased enforcement of forest regulations and restricted access to credit and output markets. The local initiative included mapping and monitoring of rural land plus promotion of economic alternatives compatible with low deforestation. The key motivation for the program may have been to reduce the costs of blacklisting. However its stated purpose was to limit deforestation, and thus we apply SCM to estimate what deforestation would have been in a (counterfactual) scenario of no local initiative. We obtain a plausible estimate, in that deforestation patterns before the intervention were similar in Paragominas and the synthetic control, which suggests that after several years, the initiative did lower deforestation (significantly below the synthetic control in 2012). This demonstrates that SCM can yield helpful land-use counterfactuals for single units, with opportunities to integrate local and expert knowledge and to test innovations and permutations on policies that are implemented in just a few locations.

## Introduction

In the absence of binding global agreements, actions taken at other scales − including national and sub-national policies, non-governmental organizations' (NGO) initiatives, and private behavior − can help to mitigate climate change. Each may have negligible climate impacts. Still, Ostrom (2010) [1] argues that collectively they provide “experimentation and experience” that inform polycentric or multi-scale climate governance. Yet to convert such actions into pragmatic learning, impacts must be understood.

Most quasi-experimental methods to evaluate impacts assume a large number of treated units, i.e., that the interventions of interest were applied to many locations. That assumption can be a serious practical constraint, given that both experimentation and optimal adjustments to local conditions often result in interventions that are unique or implemented in only a few units. In fact, decentralization has been promoted precisely because it allows local governments to tailor interventions to local conditions. This paper explains then applies one method to evaluate impacts of interventions in single jurisdictions.

Impact evaluation compares treated-unit outcomes with appropriate 'counterfactual baselines' or estimates of what would have occurred without the intervention (or ‘treatment’). Counterfactuals are never observed but are estimated using outcomes in similar units, with similar characteristics and influences over time. In differences-in-differences (DID), this is accomplished using changes over time within untreated units, which are assumed to identify the temporal influences of unobserved factors. In typical matching, this is accomplished by finding the units observationally most similar to the treated—using metrics like propensity scores—and assumed to have the same unobserved influences. Statistical testing is used to assess similarity. In comparative methods in political science [2–4], evaluations often juxtapose outcomes for a treated unit with those for one or a few other units judged to be most similar [5].

This paper describes and demonstrates a data-driven method for evaluating the impacts of treatments affecting a single treated unit − the 'synthetic control method' (SCM). SCM has been applied to unique, spatially focused events such as natural disasters [6–7], conflicts [8] and policy changes [9–10]. While no method can wish away the limitations imposed by a few treated sites and data points, SCM offers a systematic and transparent way to choose comparisons, in order to estimate impacts, with significance judged through bootstrapping or placebo tests [11].

SCM defines 'similarity' based on both observed characteristics and historical outcomes − the latter implying that unobserved influences on outcomes are also taken into account. This requires data on pre-intervention outcomes. SCM compares a weighted average of comparison units' outcomes to the outcomes over time in the treated unit. Weights are assigned to comparison units so that their combination is as close as possible to the treated unit's (with explicit weights on the characteristics and outcomes also generated by the nested optimization). Thus, SCM blends the focus on characteristics in typical matching with the focus on unobservables in DiD. SCM assumes the best fitting weighting of units in terms of pre-treatment outcomes—i.e., the synthetic cohort—would follow a time trend similar to the treated without the intervention. Since the pre-treatment outcomes were matched to define a synthetic cohort, the impact estimate for each post-treatment year is simply the difference between the treated and the synthetic outcomes.

SCM's use of the pre-treatment outcomes could improve on typical matching of characteristics. Characteristics data never include all the factors in the outcome, while the outcomes pre-treatment that SCM uses obviously reflect all influences on outcomes during the period in question. Thus, matching on outcomes as well as characteristics could, in principle, improve case selection and thus counterfactuals. For example, consider a treated unit whose deforestation rate is influenced by responsiveness to some exogenous and unobserved factor, e.g., a shift in political power that has heterogeneous local effects, through various political alliances. By matching on units' outcomes during all the pre-treatment years, SCM could identify (and weight accordingly) units with the same sensitivity to this unobserved factor.

SCM has been used to examine the effectiveness of a wide range of policies including: Arizona’s 2007 Legal Arizona Workers Act [12]; New York’s law prohibiting the use of handheld cell phones while driving [13]; Massachusetts’ 2006 health reform [14]; and the legalization of same-sex marriage in the Netherlands [15] SCM has also been used to assess impacts of European integration [16], terrorism in Ireland and Spain [17–18], (economic liberalization [19], US foreign direct investment in the United Kingdom [20], the economic impacts of the 1990 German reunification on West Germany [21], and Hurricane Iniki, which hit Hawaii in 1992 [22].

A different use of SCM has been to validate the robustness of other empirical techniques. For example, Kirkpatrick and Bennear (2014) [23] confirmed that SCM produced similar results as DiD in their assessment of the effectiveness of three California Property-Assessed Clean Energy (PACE) programs on residential photovoltaic installations. Malhotra and McCubbins (2013) [24] did a similar robustness check in their analysis of the impacts of term limits on state spending levels. We are not aware of any previous applications of SCM to land use and land-cover change (LULCC) as either the primary method or a robustness check on impact estimates.

We apply SCM to evaluate policy impacts on deforestation, a form of LULCC for which there often exist a long time series of remotely sensed data. We consider deforestation in the Brazilian Amazon, where the municipality of Paragominas, in the state of Pará, launched a “green municipality” initiative to reconcile federal limits on deforestation with local demands for economic development. The specific motivation was a federal ‘blacklist,’ or ‘embargo,’ of municipalities with the highest deforestation, established in 2007 by Decree 6.321 of the Brazilian Institute of Environment and Renewable Natural Resources (IBAMA). Blacklisting disallows rural credit from federal agencies and discourages purchases of goods produced in the blacklisted areas. That directly reduces producer incentives for deforestation. Further, municipalities have an incentive to reduce deforestation below 40 km^2^ per year to get and stay off the black list − and keep access to credit and markets. Paragominas was one of 36 municipalities on the first blacklist issued by the federal government but the only one to respond with a comprehensive local initiative, where both blacklisting and response occurred in the same “deforestation year” of 2008 based on INPE’s method of calculating annual deforestation from August of the prior year to July of the current year. The initiative included building local consensus − a ‘zero deforestation pact’ − and building capacity for local monitoring of deforestation through partnerships with NGOs and state and federal agencies.

In 2010, Paragominas became the first municipality to exit the blacklist. While INPE’s official numbers show more than 40 km^2^ of deforestation in both 2009 and 2010, Paragominas was removed from the blacklist because re-assessment of those numbers showed that they included deforestation from previous years that had previously been under cloud cover and areas of forest degradation mistaken for deforestation. According to IMAZON, the true extent of deforestation in Paragominas in 2009 and 2010 was 31 km^2^ and 35 km^2^. We do not use data from these years in our analysis, because we construct the synthetic control based on data from 2002 to 2007, and we focus on outcomes from 2011 through 2013. According to IMAZON’s deforestation estimates, Paragominas has maintained deforestation below the target level of 40 km^2^ per year ever since it was blacklisted. Given exceptionally high average deforestation from 2002 to 2007 (> 175 km^2^/year), this was a notable shift. While press coverage attributed it to the green municipality initiative, it is not clear *a priori* whether such an initiative would increase or decrease deforestation, or have no effect, relative to the blacklist by itself.

To assess the incremental impact of the initiative, we apply SCM to construct a counterfactual scenario for deforestation in Paragominas had there been no local initiative in response to blacklisting. We illustrate how SCM generates weights upon potential comparison sites − here other blacklisted municipalities − and how it facilitates consideration of the evolution of impacts over time. Our results are robust to methodological choices about the form of the dependent variable and the time frame used to develop the synthetic control. They consistently show that after three years, the initiative reduced deforestation relative to the blacklisted municipalities in the synthetic control, although this reduction was only statistically significant at the 90% level in 2012.

## Methods, Study Site and Data

### The method: Synthetic Control

**Fig 1:**
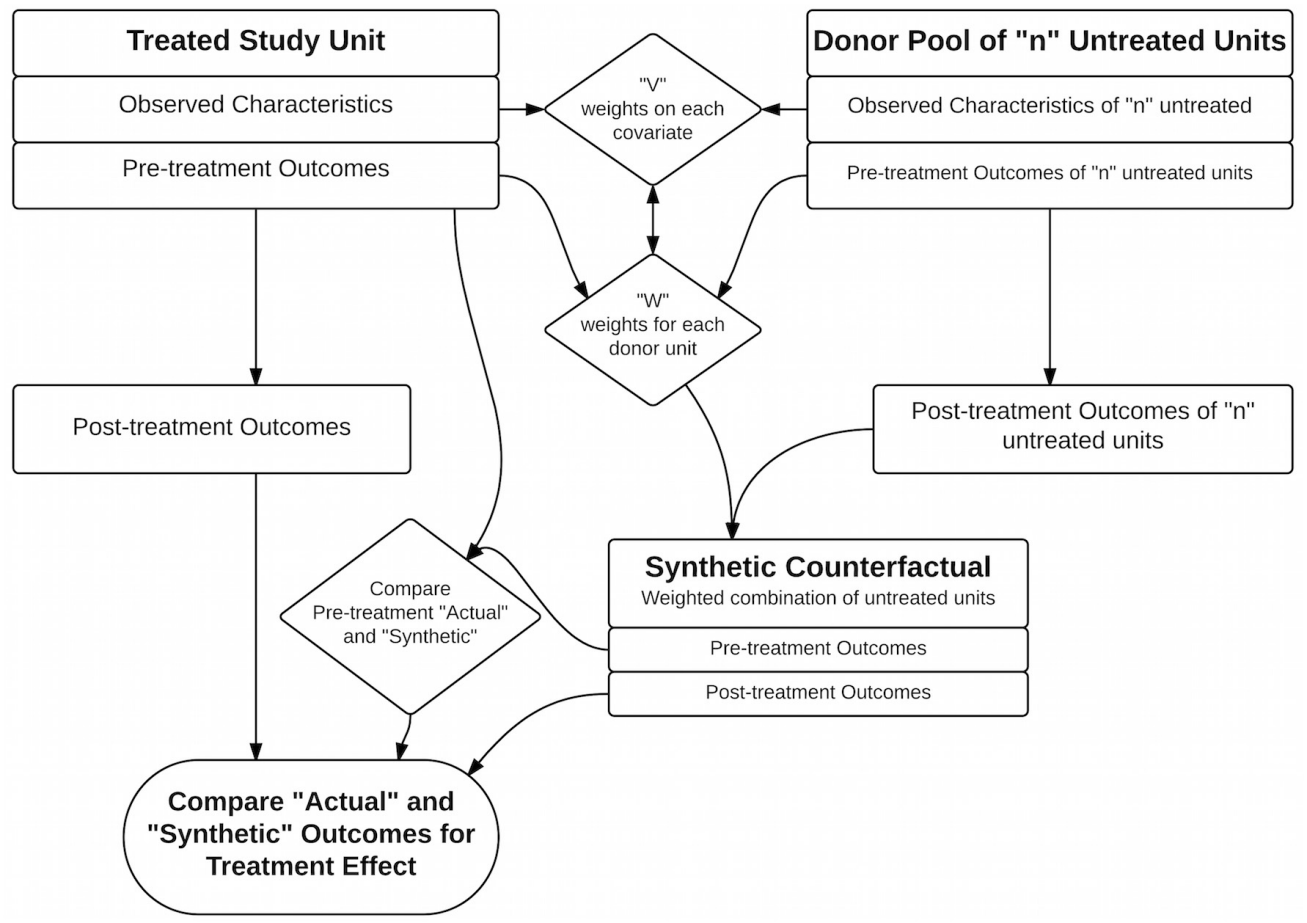
Flow diagram for SCM.

The SCM methodology was first introduced by Abadie and Gardeazabal (2003) [25] and then made available as an add-in to Matlab, R, and STATA called SYNTH [26]. Different approaches to specifying the model and statistical inference have been suggested [27]. Variations, and the software to implement them, are in active development. However, all follow the basic logic outlined in Fig 1.

SCM starts with the definition of the treated study unit (where the intervention occurred) and selection of the outcome of interest, which must be observed both pre- and post- treatment. The next step is to choose a 'donor pool', i.e., a set of potential control units judged to have some underlying structural similarity to the treated unit in terms of the processes that generate the outcome. The pool might be defined as all units in the same region, or units that are similar in terms of covariates typically used in regressions or in matching. These could include socio-economic characteristics (per capita income, transportation infrastructure, educational attainment for many outcomes), biophysical conditions (precipitation, percent riparian for land-use outcomes) and political regimes (same country or state). If the characteristics are expected to have non-linear influences, it is more important to limit the pool to units with characteristics within a reasonable range of the treated unit’s characteristics, in order to approximate linearity. Two important sets of factors to consider are: interventions or treatments other than the one of interest (e.g., in our case, the federal blacklist), and historical levels of the outcome variable (e.g., in our case, deforestation).

Given a donor pool, SCM selects weights (W) on those potential control units to define a linear combination of the control outcomes, i.e., 'the synthetic control'. These weights determine the impact estimate. For any post-treatment time period, that is the difference between this weighted average, or synthetic, outcome and the treated unit outcome. Recall that the outcome (Y) is affected by both observed (Z) and unobserved (U) factors (Y = βZ + U). For example, deforestation (Y) is driven by observed biophysical factors (Z) and unobserved political factors (U). If we searched just for similarity in Y (i.e., low WY_control_—Y_treated_), we could end up labeling as 'similar' units with quite different Z and U that just happen to balance out. Instead, SCM searches for weights W that results in both similar pre-treatment Y and similar Z, implying similar U. Since one cannot minimize all differences at the same time, but only some combination of the characteristics and outcomes differences, another vector V is required to assign weights to the variables in Z and to each year of pre-treatment Y (in the SynthR software we use, V is selected to maximize the predictive power for pre-treatment outcomes).

Thus, a synthetic control is constructed simply by implementing a minimization programmed into whatever software is used − though routines can differ in details. The quality of the synthetic control is measured by how closely the weighted synthetic outcomes match the outcomes for the treated unit in the years prior to treatment. One measure of this is the mean squared prediction error (MSPE). Best fit is always a matter of judgment, though, and might be based partly on whether a synthetic cohort seems to mirror the treated unit in terms of the turning points for outcomes plotted over pre-treatment years. If there are similar turning points, it is more likely that the treated and synthetic control units have a similar sensitivity to common factors. We give particular consideration to whether the synthetic outcome matches the actual outcome in the years just prior to treatment, because systematic error in those years could persist after treatment, biasing estimated effects. If the synthetic control passes the quality check, the final step—as shown in Fig 1—is to calculate the impact as the difference between the actual and synthetic outcomes.

Once this difference is obtained, the analyst should assess whether it is significantly different from zero. Standard statistical tests of similarity, such as of covariate 'balance', are not possible here because there is only one treated unit. Thus, other methods, such as placebo tests, must be employed to characterize the 'noise' in the estimate and thus, assess whether estimated impacts can be distinguished from that noise. One approach is to bootstrap the synthetic control by drawing sub-samples of the donor pool [28] in order to establish an empirical confidence interval for the weighted, counterfactual deforestation outcome.

### The Site: Paragominas

Understanding the impacts of local conservation innovations on LULCC in the Amazon Basin is globally important. The region contains the largest intact tropical forest landscape [29], possessing much of the world’s terrestrial biodiversity and carbon stock [30]. Rapid deforestation, starting in the 1970s, caused significant concern, prompting calls for reform of government policies that promoted deforestation (road construction, colonization projects, and agricultural subsidies). In 2004, the Brazilian government launched a Plan to Prevent and Control Deforestation in the Brazilian Amazon (or PPCD-AM), as part of a National Plan for Climate Change. In addition to reversing the legacy of perverse incentives for deforestation, the PPCD-AM also sought to address uncertain land tenure and the rise in market forces that drive deforestation, e.g., demand for cattle products and soy [31–33].

**Fig 2:**
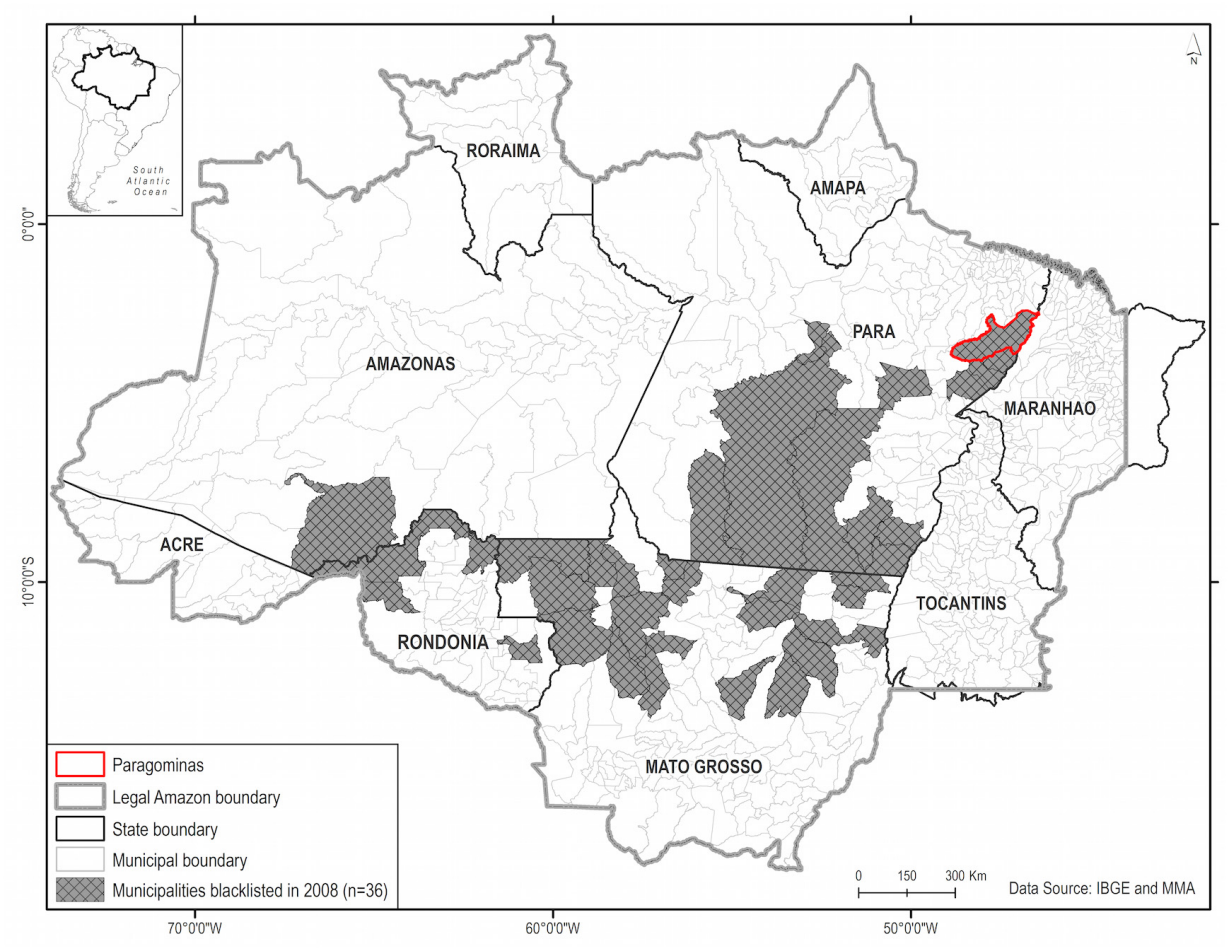
Map of the Municipalities Blacklisted in 2008.

Since 2004, deforestation in the Brazilian Amazon has declined every year but 2008 and 2013 [34]. Following the 2008 increase, Brazil's Ministry of the Environment announced a blacklist of 36 municipalities responsible for over half of the deforestation within the Brazilian Amazon (Fig 2). Producers in blacklisted (or embargoed) municipalities face greater enforcement efforts by IBAMA (the federal environmental agency), barriers to credit and finance, and limits on their access to output markets because private-sector actors have committed to avoid commodities linked to deforestation. To get off (and stay off) the “blacklist”, municipalities have to reduce annual deforestation below 40 km^2^ and to enroll at least 80% of rural properties in an environmental registry (i.e. the Cadastro Ambiental Rural (CAR)). In addition to targeting federal enforcement efforts and compliance measures, the blacklist was designed to encourage municipal governments to take responsibility for reducing deforestation − implementing the decentralization of environmental protection called for in Brazil’s 1988 constitution.

Local responses to being blacklisted varied widely. The municipality of Paragominas (1.95m ha) has been widely hailed as a success story, in the sense of decentralized protection of the environment, for declaring itself a ‘green municipality’, fostering consensus among large producers to work towards ‘zero deforestation,’ partnering with non-governmental organizations, and building local capacity to monitor and act against deforestation including implementation of the CAR [35–37]. In 2010, Paragominas was moved from the blacklist to a list of municipalities where deforestation is monitored and under control. While this clearly resulted from reductions in deforestation, a legitimate question remains: did local governance efforts influence the rate of deforestation in the municipality above and beyond the effects of blacklisting itself? This is a critical question, with significant policy implications, for anyone considering replication of the ‘Paragominas model’ elsewhere.

Paragominas in many ways typifies municipalities on the Amazonian forest frontier. Established in 1965 along the newly constructed federal highway linking Belém to Brasília (BR 316), Paragominas by 1990 was one of the leading centers for processing timber in Brazil, with over 200 sawmills [38] Cattle and agriculture—including soy—grew in the decades to follow. The result was that Paragominas was nearly 50% deforested by the time it was placed on the blacklist in 2008. The remaining forest was degraded from predatory logging, yet nonetheless there remained many sawmills, processing illegally harvested logs, which became IBAMA’s first target of enforcement action following the blacklisting. Under the ‘Arco de Fogo’ campaign in 2008, IBAMA shut down sawmills and charcoal kilns in Paragominas. That provoked a violent response from those sectors, which in turn brought an escalating federal police presence and negative press coverage to Paragominas − adding to the incentive to find some way to limit deforestation while restoring and raising production.

Starting in April 2008, the mayor of Paragominas engaged with the association of large-scale producers (via FAEPA, the Federação da Agricultura do Estado do Pará) and other local stakeholders to build consensus that the best response to blacklisting was to take greater local responsibility for LULCC. To accomplish this, the mayor established partnerships with two environmental NGOs, IMAZON and The Nature Conservancy. IMAZON provided monthly reports on deforestation, based on satellite imagery, and helped to build local capacity to use those reports to identify and respond to illegal deforestation. Both IMAZON and The Nature Conservancy supported implementation of the CAR, helping private landowners enroll as a step towards land tenure regularization. We note that these activities are complementary: the registry increases transparency and allows agencies to identify who is responsible for observed deforestation. Also helping to accomplish this, Paragominas solicited and received support from other state and federal agencies. All of this was presented as part of the ‘Paragominas green municipality’ (PGM) initiative.

### Applying SCM to the PGM

As in any evaluation, SCM requires an outcome variable. For our application, there are multiple ways to define and measure deforestation, including gross (loss of mature forest) or net (adjusted for reforestation) measures expressed in absolute (km^2^) or relative (%) terms. We focus on absolute gross deforestation, both because that is the direct target of federal and state deforestation policy (e.g., a requirement of less than 40 km^2^ annual gross deforestation to get off and stay off the blacklist) and because absolute loss translates directly into losses of ecosystem services (although both gross and net deforestation matter for these services). Because municipalities in the Brazilian Amazon vary immensely in terms of size, though, we also consider deforestation as a percent of municipal area. Both outcome measures are derived from data provided by INPE (the Brazilian National Space Agency) and can be evaluated for single years or summed across years.

We illustrate SCM by estimating how much deforestation would have happened in Paragominas had it not responded to blacklisting by creating the PGM initiative. However we emphasize that, locally, economic outcomes are very important too. From that local perspective, the core goal of PGM was to allow continued production within the constraints of federal restrictions on deforestation. Thus, economic outcomes, such as municipal GDP or tax receipts, should be examined in future research.

**Fig 3:**
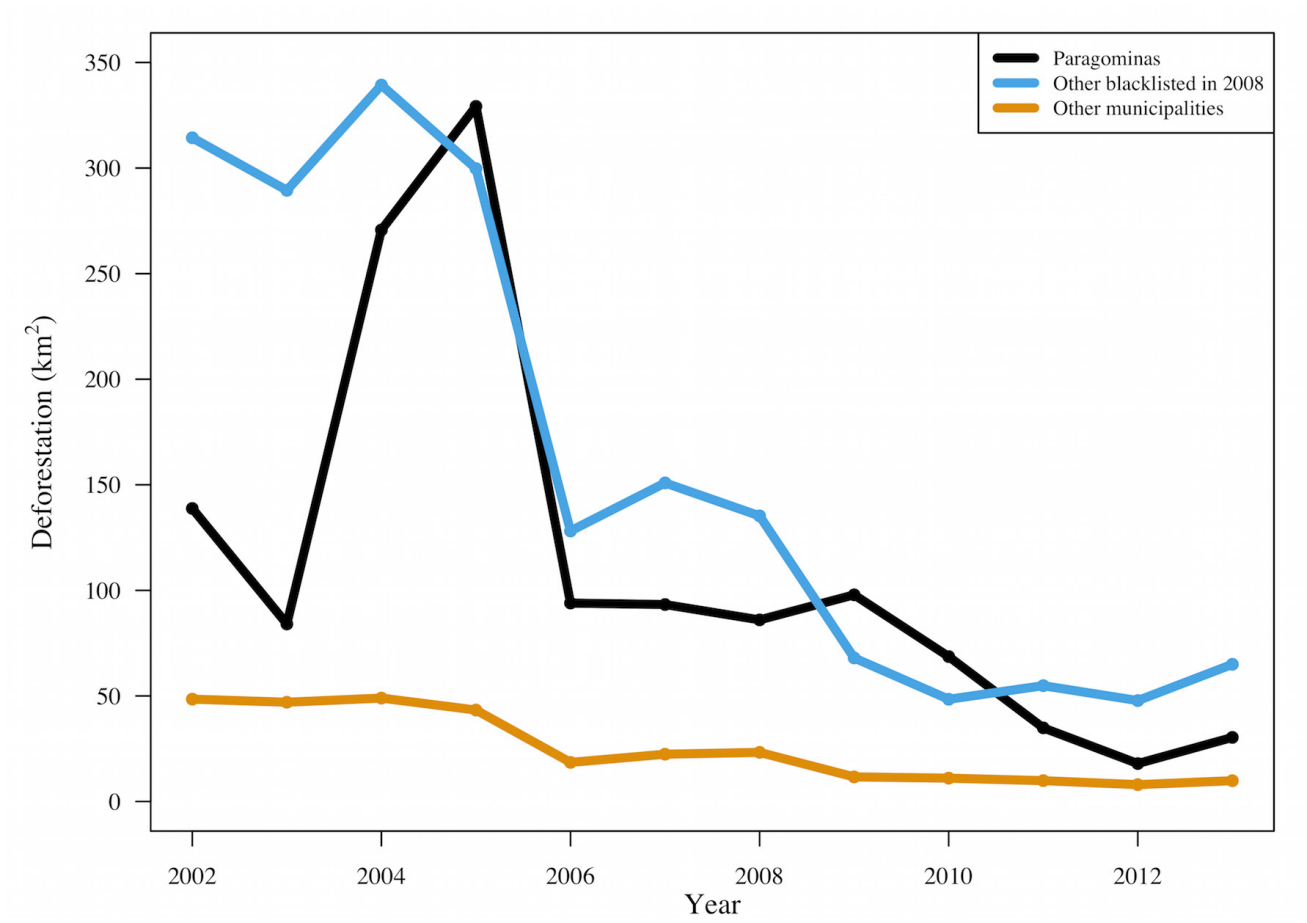
Average Annual Deforestation (KM^2^) per Municipality.

Fig 3 compares annual deforestation from 2002 to 2013 in Paragominas to the averages both for all other municipalities blacklisted in 2008 and for all other municipalities in the Brazilian Amazon. The clear drop in deforestation following the 2004 federal policy shift suggests the influence of federal interventions. Deforestation also fell after municipalities were blacklisted, although not consistently in every year. In the first two years after blacklisting, the official deforestation statistics from INPE show that deforestation held steady in Paragominas while it dropped in other blacklisted municipalities, but those statistics have been questioned because low cloud cover may have revealed deforestation that had occurred previously and because forest degradation may have been misclassified as deforestation. Since 2010, deforestation in Paragominas has been below the average deforestation of the other municipalities that were blacklisted in 2008. While Fig 3 shows that the initiative was associated with reduced deforestation in Paragominas, it does not show causality because it does not take into account differences between municipalities. Following the logic of impact evaluation, to estimate causal impacts we need to compare the outcomes in Paragominas with an appropriate counterfactual based on similar municipalities. That brings us to synthetic controls constructed to match municipalities' characteristics and pre-treatment outcomes.

In defining the donor pool on which SCM draws, qualitative judgment about similarity inevitably enters. Since the federal blacklisting of municipalities is widely believed to be carefully targeted and to have significant influence [39–40], we focus on a pool of municipalities that were blacklisted at the same time as Paragominas. This means we evaluate the impact of this local initiative *conditional* on the federal policy (as compared to the alternative of evaluating the *combined* impact of the blacklist and the local effort, compared to a donor pool of municipalities not on the blacklist).

For fitting pre-treatment outcomes, a nearly 15-year, unbroken record of remotely sensed annual deforestation for all municipalities in the Brazilian Amazon is available from INPE. Acknowledging the federal government’s new effort to reduce deforestation starting in 2004, we treat before and after 2004 as distinct time periods in terms of the structural drivers of deforestation [41]. Focusing on 2004–2007 could best reflect the deforestation processes 'in the shadow' of the key federal intervention. Yet fitting from 2002 might better identify municipalities that are good matches in terms of underlying local deforestation processes that are also helpful comparison units to use over the longer run. We construct synthetic controls with both periods in order to comment on the sensitivity of our results to this choice. Because the PGM was launched in April 2008, we exclude 2008 from both our fitting and our impacts years. Fig 2 shows six years (2002 to 2007, inclusive) of deforestation data before the PGM “treatment” in 2008 and five years after the “treatment” (2009 to 2013, inclusive), although two of those years have known data quality problems (2009 and 2010).

Finally, we choose observable characteristics (Z) to include in constructing synthetic controls. Typical matching considers only such Z and tests their balance. Within SCM, which also makes use of outcomes, recall that matching on Z as well as Y helps identify unobservable factors that drive outcomes (if Y = BZ + U, similar Y and Z imply similar U). While it is possible to implement SCM with information only on Y, we construct the synthetic controls based upon similarity in both summary outcome statistics, specifically average annual deforestation in 2002–2003 and 2004–2007, and structural drivers of deforestation, selected based on the empirical literature about deforestation drivers in the Amazon. Specifically, our Z vector includes time-invariant characteristics (mean slope, mean elevation) and pre-intervention characteristics (road density, % indigenous reserves, % federal conservation units, % federal land reform settlements, % under IBAMA embargo, rural properties density, % with significant land tenure conflicts as reported by the Catholic Church (CPT), number of years free of hoof and mouth disease, indicator for a slaughterhouse, density of cattle, indicator for mining, per capita GDP and agricultural GDP, % GDP from agriculture, share of income earned by top 10%, population density, education (as included in Human Development Index), and whether the mayor elected in 2008 was in the same party as the state governor (PSDB)).

Given all that, SCM is purely mechanical. In the 'Synth' package for R that we employ, a nested optimization routine picks weights V on all the Z and the past Y (Abadie uses X to designate all Z and Y). For instance, one can start Synth off with regression coefficients from a model of deforestation as the V then, given V, weights W on municipalities that define the synthetic cohort are chosen to minimize the difference between synthetic control (WX) and X for Paragominas. V is then adjusted to improve this fit.

In our case, V includes weights on the deforestation averages for the two time periods (past Y) and on the characteristics of municipalities (Z). These weights vary substantially depending on whether the deforestation outcomes for the entire pre-treatment period or for just 2004–2007 are considered. Using the entire period, V has most weight on the percent of municipality in conservation units, percent with land conflicts, and whether the most recently (pre-treatment) elected mayor was politically aligned with the governor. Using just 2004–2007, V has most weight on population density and percent of GDP from agriculture. All of these factors are clearly relevant to deforestation, but SCM weights them very differently depending on the time period. The vector W also varies depending on the pre-treatment fitting period. Using the entire period, the W gives 86% of the weight to two municipalities (Paranaita and Santana do Araguaia) and lower weights to four other municipalities. Using just 2004–2007, roughly one-third of the total weight in W is assigned to each of three municipalities: Machadinho d’Oeste, Rondon do Pará, and Santana do Araguaia. Only one municipality (Santana do Araguaia) is given significant weight in both of these synthetic controls. This is grounds for legitimate debate and research but at least is transparent.

Plots of annual deforestation (in KM^2^) in Paragominas and the synthetic control allow us both to assess the quality of the synthetic control (by comparing pre-treatment deforestation in the synthetic and in Paragominas) and to evaluate the impact of the initiative (by comparing post-treatment deforestation in the synthetic and Paragominas). To assess whether the estimated impacts in each year are significantly different from zero, we also need an estimate of the noise (or error) in our estimates. We accomplish this with bootstrapping, constructing 1,000 bootstrapped synthetic controls from donor pools of 25 municipalities drawn at random (without replacement) from the full donor pool. Using the W weights for each of these synthetic controls, we plot the 5^th^, 50^th^ (median) and 95^th^ percentile of the synthetic deforestation outcomes in each year. The median deforestation of the bootstrapped synthetic controls is very close to the deforestation in the synthetic control constructed from the full donor pool. We therefore only portray the 5^th^, 50^th^ (median) and 95^th^ percentile synthetic controls in the plots.

## Results

**Fig 4:**
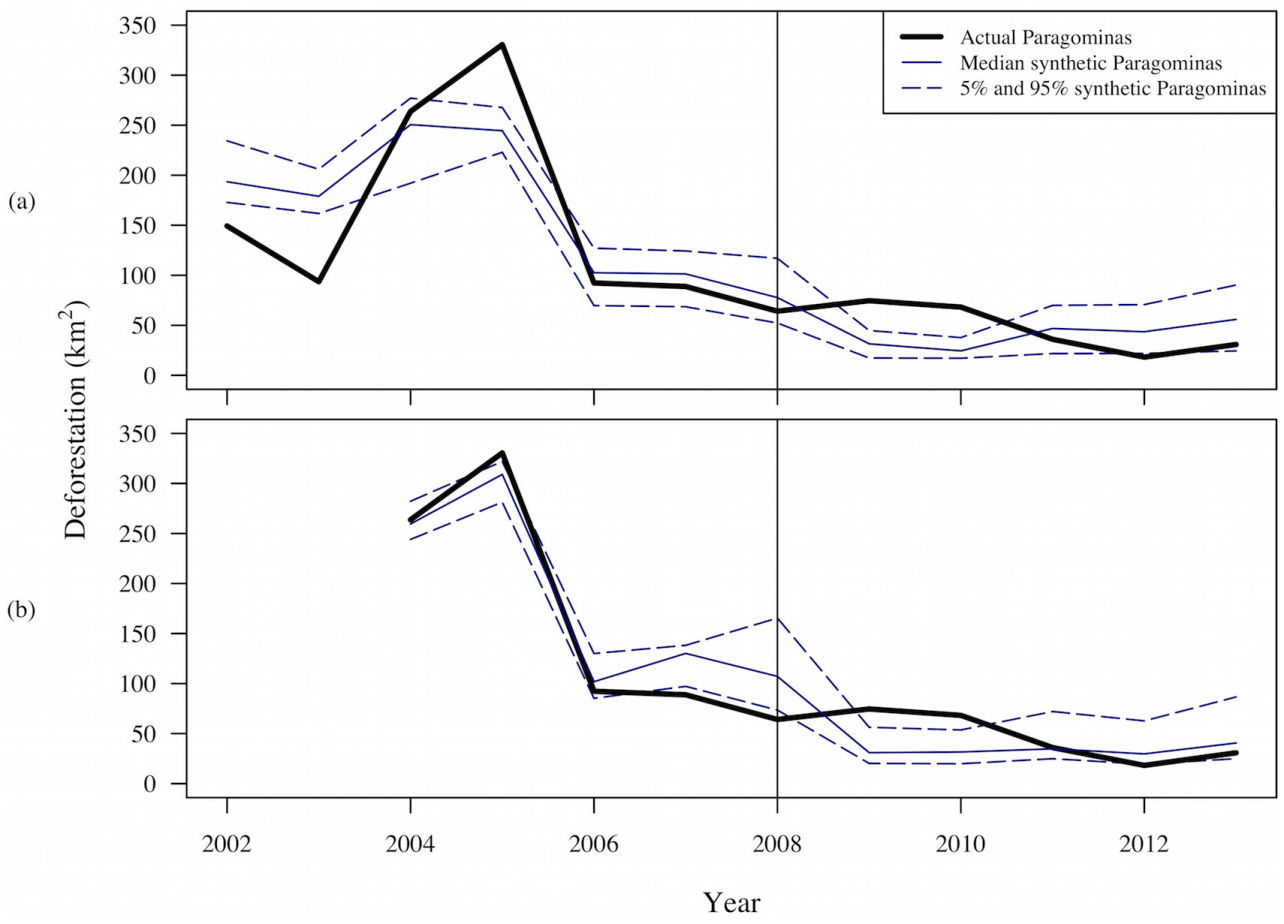
Deforestation (KM^2^) in Paragominas and Synthetic Control constructed from 2002–2007 and from 2004–2007 Deforestation.

Because concerns about how INPE distributes deforestation observed in areas that had been under cloud cover in previous years particularly affect 2009 and 2010, we focus on findings after Paragominas was taken off the blacklist in 2010. From 2011 through 2013, deforestation in Paragominas was lower than at any time in the previous decade and lower than the median counterfactual deforestation predicted by the synthetic control constructed from other municipalities blacklisted at the same time. In 2012, deforestation in Paragominas was below the 5^th^ percentile of deforestation in the bootstrapped synthetic controls, which we interpret as significantly different from zero. Specifically, Fig 4a shows that only 3% of the synthetic controls predict less deforestation than actually occurred in Paragominas in 2012. Deforestation rose in Paragominas—and throughout the Brazilian Amazon—in 2013, but it remained below most (>90%) of the counterfactual estimates from the bootstrapped synthetic controls.

These results are based on synthetic controls that closely track actual deforestation in Paragominas during the pre-treatment years, 2002–2007 (Fig 4a and 4c). Both the actual and the synthetic counterfactual deforestation peak in 2004 or 2005, fall in 2006, and then stabilize. Actual deforestation in Paragominas falls within the 90% confidence interval of deforestation in the bootstrapped synthetic controls in the year of treatment (2008) and two previous years. Somewhat surprisingly, the synthetic control created with data from only 2004 to 2007 (Fig 4b) does not match actual deforestation in those as well, with actual deforestation outside the 90% confidence interval in all years except 2004 and 2006. However, all of the synthetic controls are clearly improvements on the average of all blacklisted municipalities shown in Fig 3.

**Fig 5:**
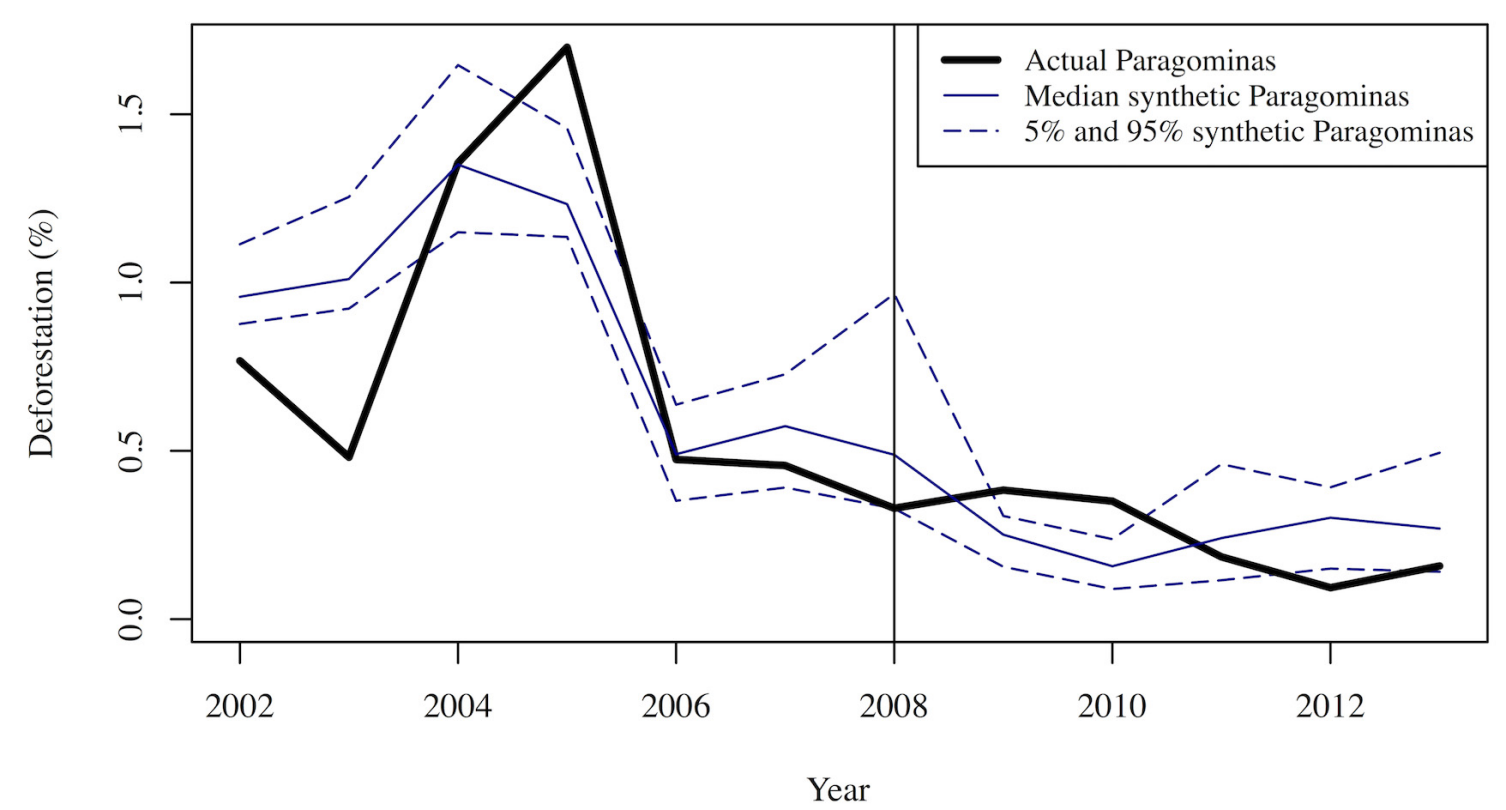
Deforestation (% of Municipality) in Paragominas and Synthetic Control constructed from 2002–2007 Percent Deforestation.

One other important robustness check is to construct the synthetic control and evaluate impact in terms of deforestation as a percent of the municipality. In this case, there are once again differences in the V and W matrices, with the greatest weights place on the political party of the mayor and an indicator of whether there is a slaughterhouse operating in the municipality. The W matrix places 82% of the weight on just two municipalities: Altamira and Porto Velho, a completely different set than selected in the previous analysis. Nevertheless, Fig 5 shows similar results: deforestation in Paragominas initially holds steady but is substantially lower in 2012 and near the lower end of the 90% confidence interval in 2013. Thus, these findings seem to be quite robust, not varying with analyst choices about the form of the outcome variable or the time period for the analysis or with the specific combination of municipalities selected by SCM.

## Discussion

Our results suggest that the PGM initiative may have a lagged impact on reducing deforestation, above and beyond any impact of the blacklist. However, because this effect is only significantly different from zero in 2012, final judgment about the effectiveness of the initiative is contingent on future years of deforestation data to confirm whether the impact is durable. One possibility is that the federal blacklist left little room for influence by other initiatives. This is consistent with the finding by Assunção and Rocha (2014) [42] that the primary mechanism for the blacklist’s impact was increased enforcement by the federal environmental agency, rather than engagement of local governments and stakeholders in deforestation control. In this context, it is also worth noting that the PGM did not increase deforestation relative to municipalities where there was no organized local response—despite the fact that a key objective was to restore economic activity within the constraints of the blacklist. That raises the question of whether the PGM did in fact increase economic activity relative to the counterfactual, which could also be evaluated with SCM, given time series data on economic outcomes.

Our evaluation of the PGM illustrates how SCM provides a statistical approach to choosing comparison cases for impact evaluation in low-N settings, e.g. pilot projects or locally tailored interventions to reduce deforestation. In such settings, comparative methods used in many social science disciplines have typically relied on expert judgment to select a single comparison case. When expert judgment cannot identify a single comparison unit similar to the intervention unit, common alternatives include a simple or weighted average of the available comparison units. SCM provides a transparent and justifiable means to optimize the selection and weighting of comparison units and improve on the development of counterfactuals for impact evaluation. Comparison of Figs 3 and 4 demonstrates that in the case of Paragominas, the SCM weighted average of selected units mirrors pre-intervention trends much more closely than the average of all blacklisted municipalities in the donor pool. This is true despite the fact that we have already imposed an important qualitative restriction by limiting the donor pool to municipalities blacklisted in the same year as Paragominas. By using SCM to select (and weight) just a few municipalities from the donor pool, we are able to match both the level of deforestation (with low MSPE) and the trends in pre-intervention deforestation, which were quite variable in the years leading up to 2008. The pre-treatment fit thus achieved is assumed to be indicative of the post-treatment fit of the synthetic control to the true counterfactual.

True counterfactuals are never observed and thus cannot be compared to the synthetic control. However, we believe that SCM produces a credible counterfactual, arguably more credible than matching on just the characteristics of the municipalities or selecting a few municipalities based on expert judgment. While SCM is not a uniquely defensible way to pick comparison units, it is both standardized and transparent, as we can see and judge the weights placed on different covariates and municipalities. In the case of the synthetic control for Paragominas, the optimization routine placed large weights on relatively few covariates with small weights on the others, and it placed positive weights on only a few of the municipalities. This makes it pragmatic to discuss those weights with local experts, to assess whether they make intuitive sense in terms of the individual units and combinations of units that are picked. Expert judgment could be further integrated by setting weights on covariates and/or ruling municipalities in or out of the donor pool.

Both the time period and the specific form of the outcome measure used to construct the synthetic control affect the V and W weights, although the end results are highly robust in our case study. Specifically, regardless of these choices, we find that PGM significantly reduced deforestation only in one year (2012), although deforestation in Paragominas was also below the median deforestation in the bootstrapped synthetic controls in both 2011 and 2013. The ability to examine how outcomes evolve over time is another advantage of SCM relative to conventional matching approaches, which estimate average impacts at a particular point in time. With SCM, we obtain both numerical estimates and an intuitive figure showing how an outcome such as deforestation evolves over time under both actual and counterfactual conditions.
